# A Tunable Strain Sensor Using Nanogranular Metals

**DOI:** 10.3390/s101109847

**Published:** 2010-11-02

**Authors:** Christian H. Schwalb, Christina Grimm, Markus Baranowski, Roland Sachser, Fabrizio Porrati, Heiko Reith, Pintu Das, Jens Müller, Friedemann Völklein, Alexander Kaya, Michael Huth

**Affiliations:** 1 Physikalisches Institut, Goethe Universität, Max-von-Laue-Str. 1, 60438 Frankfurt am Main, Germany; E-Mails: c.grimm@physik.uni-frankfurt.de (C.G.); baranowski@physik.uni-frankfurt.de (M.B.); sachser@physik.uni-frankfurt.de (R.S.); porrati@physik.uni-frankfurt.de (F.P.); das@physik.uni-frankfurt.de (P.D.); j.mueller@physik.uni-frankfurt.de (J.M.); michael.huth@physik.uni-frankfurt.de (M.H.); 2 Nanoscale Systems GmbH, Robert-Bosch-Str. 7, 64293 Darmstadt, Germany; E-Mail: kaya@nanoss.de (A.K.); 3 RheinMain University of Applied Sciences, Am Brückweg 26, 65428 Rüsselsheim, Germany; E-Mails: reith@imtech-fhw.de (H.R.); friedemann.voelklein@hs-rm.de (F.V.)

**Keywords:** cantilevers, electron beam induced deposition, granular metals, strain sensors

## Abstract

This paper introduces a new methodology for the fabrication of strain-sensor elements for MEMS and NEMS applications based on the tunneling effect in nano-granular metals. The strain-sensor elements are prepared by the maskless lithography technique of focused electron-beam-induced deposition (FEBID) employing the precursor trimethylmethylcyclopentadienyl platinum [MeCpPt(Me)_3_]. We use a cantilever-based deflection technique to determine the sensitivity (gauge factor) of the sensor element. We find that its sensitivity depends on the electrical conductivity and can be continuously tuned, either by the thickness of the deposit or by electron-beam irradiation leading to a distinct maximum in the sensitivity. This maximum finds a theoretical rationale in recent advances in the understanding of electronic charge transport in nano-granular metals.

## 1. Introduction

Granular metals are artificial materials in which a conducting phase made of metallic nanoparticles is (randomly) dispersed into an insulating matrix. In between neighboring metallic nanoparticles a tunnel coupling *g* exists, which can be introduced as a dimensionless quantity measured in units of the quantum conductance 2 *e^2^/h*. A material is considered granular if the intragrain conductance g_0_ greatly exceeds the intergrain tunnel coupling *g.* Its electronic properties can be adjusted, leading to an important role in fundamental studies as well as nanotechnological applications [1,2]. The charge transport in such systems is dominated by tunneling between neighboring metallic nanoparticles, a process that is strongly influenced by correlation effects, such as the Coulomb blockade in the limit of weak intergrain coupling, which has a direct bearing on the conductivity. These fundamental processes can be used for practical applications, since the tunnel coupling has an intrinsically exponential dependence on the inter-grain distance that is altered under strain, therefore making nanogranular metals suitable for strain-sensing applications [2–5].

The fields of micro- and nanoelectromechanical systems (MEMS and NEMS) as enabling technologies for sensor device development are rapidly progressing, due to the increasing demand for a continuous down-scaling of sensor functions in a broad variety of different application fields. Different approaches have been followed for nano- and microscale strain/stress measurements ranging from well-established methods, e.g., optical and piezoresistive (see Reference [6,7] and references therein), to methods still being in their infancy, e.g., carbon nanotubes (CNT) [8], nanowires (NW) [9], and diamond like carbon films (DLC) [10]. In this paper, we present a novel methodology for strain sensing based on nanogranular metals using Pt-based deposits as a particularly case study. A specific strength of this methodology is that it does not entail complex fabrication procedures and is readily adaptable to various sensor applications. The process is a maskless bottom-up technique that can be applied to nearly all surfaces leading to a wide area of possible applications. In addition, the high resolution of the focused electron-beam-induced deposition (FEBID) technique allows for easy downscaling of sensor structures to below 100 nm. The strain sensors show a highly linear response in deflection measurements that were performed on a cantilever template. The gauge factor for these nano-granular metals depends on the conductivity of the sensor, which can be tuned by electron-beam irradiation leading to a distinct maximum in the sensitivity that can be attributed to a persistent change of the dielectric carbon matrix of the Pt-sensors under electron-beam exposure. By *in situ* electrical conductivity measurements we are able to tune the sensitivity of the sensor. We find the noise of the sensor elements to follow a 1/*f*-frequency dependence hitting the Johnson noise level at about 1 kHz and suggest possible applications.

## 2. Experimental Section

**Figure 1.**
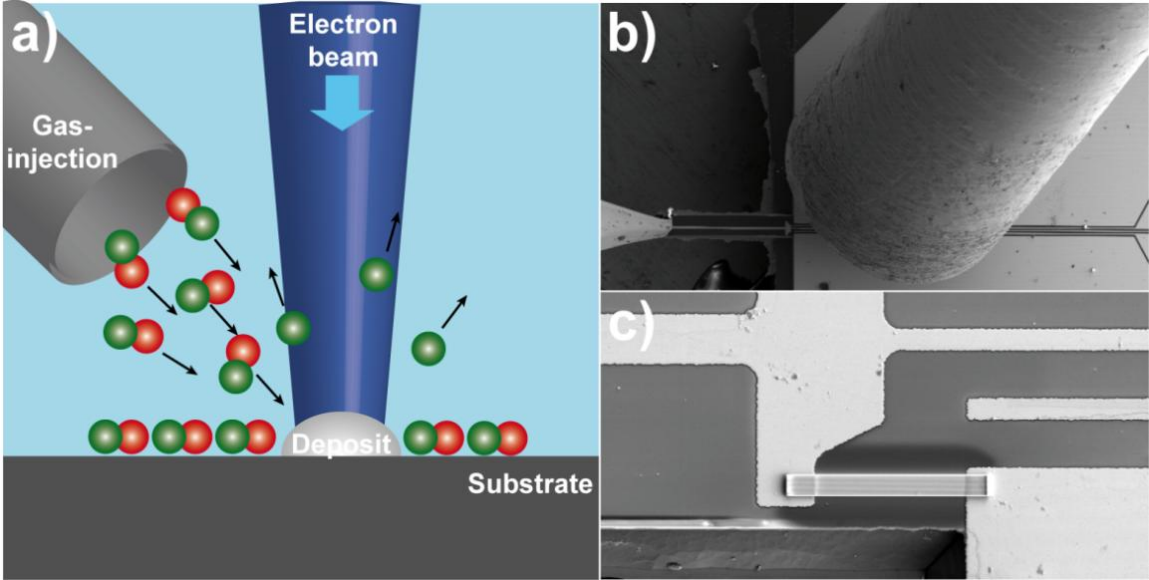
**(a)** Schematic of the FEBID process. The precursor gas is introduced via a gas injection system in close proximity to the focus of the electron beam. The electron beam dissociates the precursor molecules forming the nano-granular deposit; **(b)** SEM image of the experimental setup showing cantilever, gas injection capillary (right), and nanomanipulator needle (left); **(c)** SEM image of deposited sensor structure on the cantilever.

The strain sensor was fabricated by FEBID of the precursor trimethylmethylcyclopentadienyl platinum [MeCpPt(Me)_3_] using a dual-beam SEM/FIB microscope (FEI, Nova Nanolab 600) equipped with a Schottky electron emitter and an ultimate resolution of 1 nm. The microscope is equipped with a gas injection system, which introduces the precursor gas via a 0.5 mm diameter capillary in close proximity to the focus of the electron beam [11]. A schematic of the experimental setup is shown in Figure 1. The sensor structures were grown on a cantilever-substrate that was pre-patterned with 120 nm thick Au/Cr-contacts defined by UV photolithography. During the growth of the sensor elements their conductance was measured *in situ* at a fixed bias voltage using a source meter (Keithley 2400). Details of the setup can be found elsewhere [12]. The deflection measurements were performed with a closed-loop nanomanipulator (SmarAct SLC1720-S) that was integrated inside the electron microscope. During the deflection measurements the relative change in conductance due to the deflection was measured using a lock-in amplifier (Stanford Research SR830) and a Wheatstone bridge setup. Low-frequency resistance fluctuations have been measured by a standard ac technique [13] in a five-terminal setup, where the sample is placed in a Wheatstone bridge in order to suppress the constant voltage offset. The output signal of a lock-in amplifier (SR830), operating at a driving frequency *f*_0_ of typically 137 Hz to 517 Hz, was processed into a spectrum analyzer (SR785) by means of a low-noise preamplifier (SR560), which was preferably operated near the eye of its noise figure.

**Figure 2.**
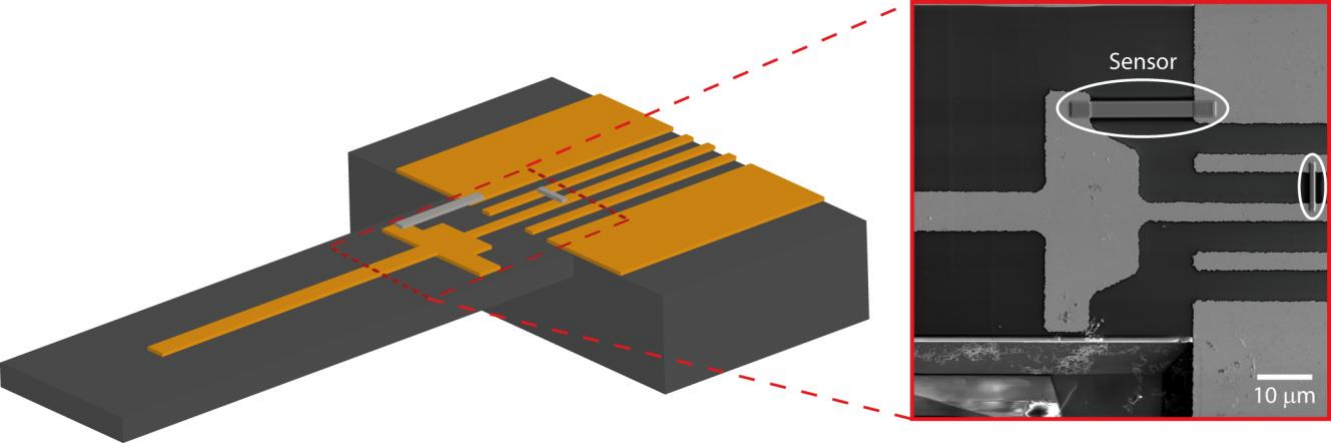
3D-schematics of the cantilever chip used for strain sensor measurements. The cantilever dimensions are length: 500 μm, width: 70 μm, height: 10 μm. The strain sensors are deposited between the Au/Cr-contacts across the bending edge. The inset displays a SEM image of the substrate for two Pt-deposits, one serving as the strain sensor and one for reference.

Figure 2 displays a schematic of the cantilever chip used for the strain sensor measurements. For the probe fabrication the starting material is a SOI wafer with 300 nm thick super low stress Si-nitride layers on both sides of the polished SOI wafer. The 10 μm thick top Si layer is separated from the bulk Si by a 600 nm thermal SiO. After pre-patterning of 120 nm thick Au/Cr-contacts by UV photolithography, the cantilever was prepared by a combination of reactive ion etching and anisotropic Si etching using KOH solution. The chip is equipped with five Au/Cr-contacts. Four of them are at the bending edge of the cantilever, whereas the center-electrode reaches onto the cantilever. This setup allows for the writing of a full Wheatstone bridge assembly. The FEBID structures were deposited between the Au/Cr-contacts. The structure for strain-sensing was deposited across the bending edge of the cantilever. It is important to point out that the cantilever template was chosen for simplicity of measuring the gauge factors, but our sensor is not limited solely to cantilever applications.

The inset of Figure 2 shows a SEM image of two FEBID structures written between the Au/Cr-contacts. The structures shown were prepared with an electron beam energy of 5 keV and an electron current of 1.6 nA using stripe-like patterns that were repeatedly rastered over the structure at fixed dwell time per pixel and pitch between pixels. During the growth of the sensor structures *in situ* conductivity measurements were performed so that the targeted conductivity of the sensor could be monitored (Figure 4(b)). After the deposition energy-dispersive x-ray analysis (EDX) at 5 keV electron beam energy was performed in order to determine the material composition of the deposit. For all investigated deposits the sensor material was composed of 22–23 at% Pt inside a carbon matrix.

## 3. Results and Discussion

### 3.1. Strain Resistance Effect

**Figure 3.**
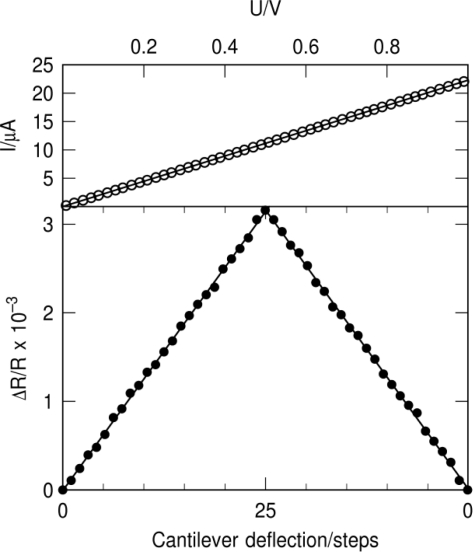
**Top:** Exemplary I(V)-characteristic for deposited strain sensor; **Bottom:** Exemplary Δ*R/R vs.* cantilever deflection curve used for measuring the deflection sensitivity. One deflection step corresponds to a deflection of the cantilever of 164 nm.

In order to measure the sensitivity of the sensor structures deflection measurements on a cantilever template were performed (Figure 2). The deflection sensitivity for a rectangular cantilever beam, which relates the relative resistance change Δ*R/R* to the cantilever deflection Δ*z* can be expressed as:

(1) $$\left(\frac{\Delta R}{R}\right)\frac{1}{\Delta z}=K\frac{3\left(l-\frac{1}{2}L\right)t}{2l^{3}}$$

where *K* is the gauge factor of the Pt-sensor, *l* is the cantilever length, *t* is the cantilever thickness and *L* the sensor length [14]. We measured the sensitivity of our Pt-sensors by deflecting the cantilever of the sensor chip with a closed loop nanomanipulator while measuring the relative change in resistance as a function of cantilever deflection. Figure 3 (bottom) displays an exemplary sensitivity measurement for the strain sensor where the cantilever was deflected and relaxed again. Before and after each deflection measurement the I(V)-characteristic of the sensor was taken. As can be seen in Figure 3 (top), the I(V)-characteristic shows a highly linear behavior. We could follow this linearity up to voltages of at least 5 V corresponding to an electric field of 2.5 kV/cm.

### 3.2. Gauge Factor Measurements

Taking into account an analysis scheme developed by one of us (M.H.) [2] that allows for a quantitative description of the sensitivity of nano-granular metal based strain sensors, the parameters that influence the gauge factor most are the intergrain conductance *g*, the grain size of the metallic nanoparticles *d*, and the dielectric constant of the insulating matrix *ε_i_*. The FEBID process allows to manipulate these parameters, thus influencing the electronic transport properties, in particular close to the metal-insulator transition [15,16]. For our nanogranular deposits we are still on the insulating side of the metal-isolator transition. There the model predicts for an exemplary parameter set deduced from experiments with Au nanocrystals a distinct maximum for the gauge factor as a function of intergrain conductivity in the Arrhenius transport regime. The main cause for the predicted maximum in the gauge factor is the thermally activated behavior of the electrical conductivity that is governed by the Mott gap Δ*_M_* as activation energy. The Mott gap depends exponentially on the intergrain tunnel coupling strength leading to an analytical maximum in the gauge factor. In the next step of our experiment we were specifically looking for evidence of the predicted behavior.

**Figure 4.**
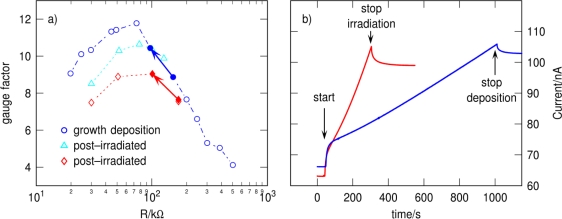
**(a)** Measured gauge factors for Pt deposits during growth deposition (circles) and for post-irradiated deposits (triangles, diamonds). The two arrows indicate the displayed *in situ* conductivity measurements as a function of time during deposition (blue arrow) and post-irradiation (red arrow) shown in (b); **(b)** Conductivity measurements during deposition (blue) and post-irradiation (red) for a bias voltage of 10 mV.

Our ability to measure the conductivity of the Pt deposits during deposition enables us to systematically study the influence of the conductivity on the sensitivity of the sensor. In a first experiment we changed the conductivity by gradually increasing the thickness of the Pt deposits and therefore increasing the volume of the conducting material. Figure 4(a) shows the measured gauge factors for different conductances of the Pt-sensors corresponding to various thicknesses (open blue circles). We observe a clear maximum in the gauge factor at ∼75 kΩ, which corresponds at this point to a resistivity of ∼0.34 Ωcm of the sensor material. At this point of highest sensitivity we measured a temperature coefficient of resistivity (TCR) of ∼−5,500 ppm/K at 280 K.

The conductivity of the Pt deposits does not only change with increasing thickness but can also be altered by the electron-beam itself during the deposition due to irradiation effects that influence the grain size of the metallic nanoparticles and/or the dielectric constant of the insulating matrix. At 5 keV beam energy the penetration depth of the electrons into the sensor material amounts to about 120 nm as deduced from Monte Carlo simulations [17]. This implies a continuous electron irradiation of the existing deposit as its thickness increases. For the Pt precursor Pt(PF_3_)_4_ electron post growth irradiation has been studied [18]. These experiments showed that the conductivity of the Pt deposits decreased under electron beam irradiation accompanied with an increase of the platinum nanocrystallite grain size as well as a volume reduction of the deposits, presumably due to the loss of phosphor and fluorine from the insulating matrix.

To study the influence of focused electron-beam irradiation on the Pt deposits, made from the phosphor and fluorine free precursor used here, and the resulting gauge factors we deposited two Pt-sensors and terminated the growth at a resistance of ∼170 kΩ and ∼130 kΩ, respectively. After the deposition we switched off the precursor gas flux and waited for more than two hours to guarantee that no residual precursor molecules were present in the chamber. Before starting the irradiation experiment we measured the gauge factors of the sensors. As can be seen in Figure 4(a), the starting gauge factors are in excellent agreement with the thickness dependent gauge factors indicating the high reproducibility of the fabrication procedure using the precursor [MeCpPt(Me)_3_]. Afterwards we irradiated the deposits with the electron-beam using the same parameters as for the previous deposition only without the precursor gas flux until the targeted conductivity was reached and again measured the sensitivity of the sensors. We followed the change of the conductivity over time during the irradiation (Figure 4(b) red curve) between two deflection measurements marked by the red arrow in Figure 4(a). It shows a much steeper increase in the conductivity as compared to the conductivity measurement during the growth of the sensors (Figure 4(b) blue curve). This behavior can be understood, if one assumes that the electron-beam induced changes in the previously formed deposit lead to a more pronounced conductivity increase than does the simple increase in deposit height. The measured gauge factors for both post-irradiated sensors also show a distinct maximum with a slightly reduced value depending on the starting conductivity before irradiation. At this stage we speculate that the main influence of the electron irradiation is to change the insulating matrix such that the intergrain coupling strength increases. The observed maximum would then be evidence for the theoretically predicted maximum in gauge factor due to the exponential dependence of the Mott gap on the coupling strength [2]. The impact of electron-beam irradiation on the carbon matrix can be analyzed using visible light Raman Spectroscopy, from which the composition of the carbon matrix can be deduced [19]. The experiments indicate an observable change of the carbon matrix from a more amorphous type to a more nanocrystalline type under electron-beam irradiation [20] aiding the observed change in conductivity. Whether also the Pt grain size is altered under radiation has yet to be determined by transmission electron microscopy of our Pt-sensors. Recent experiments for [MeCpPt(Me)_3_] indicate no significant change in the grain size of the Pt nanocrystallites under electron-beam irradiation [21].

One major aspect for sensor devices is long-term stability under ambient conditions. Even after several weeks in air our Pt-sensors show only a small increase in conductivity and still have high gauge factors. This small change in conductivity can be further suppressed by passivation of the sensor, e.g., with SiO_2_, an issue that is being addressed in current experiments.

### 3.3. Noise Measurements

**Figure 5.**
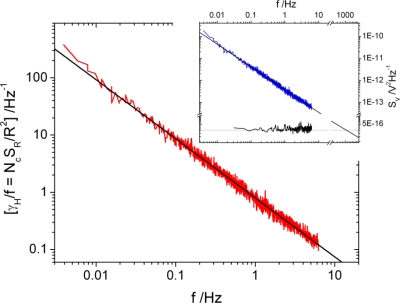
Normalized resistance noise power spectral density (PSD) of a Pt-sensor with dimensions 15.1 μm × 1.3 μm × 0.28 μm taken at room temperature. Inset: Voltage noise PSDs taken at currents *I =* 38.8 μA (blue) and *I =* 0 (black). The black curve corresponds to thermal noise, whereas the blue curve can be fitted to *S* ∝ 1/*f^α^* with a slope α = 1.03.

In order to estimate the resolution limits of the sensor, noise measurements were performed. Figure 5 shows the normalized resistance noise power spectral density (PSD) of a Pt-sensor with dimensions 15.1 μm × 1.3 μm × 0.28 μm taken at room temperature. The spectra are of generic 1/*f*-type. Following the empirical description of 1/*f*-noise first given by Hooge [22]: *S_R_* = *γ_H_R*^2^/*n_c_*Ω*f*, where *γ_H_* is a dimensionless parameter referring to the noise level of the system [23], *n_c_* the concentration of charge carriers and Ω the noisy volume, the data are scaled to the total number of charge carriers in the noisy volume *N_c_* = *n_c_*Ω. From this representation one can read from the data at *f* = 1 Hz a Hooge parameter γ*_H_* of about 0.8, which is in good agreement with values found in earlier studies of Pt metal-insulator composites prepared by co-evaporation of Pt and Al_2_O_3_ [24]. As observed in these studies, our Pt-metal-insulator composite sample is also characterized by substantial resistivity fluctuations in the low-frequency regime. Experiments are in preparation to study the temperature dependence of the 1/*f*-type fluctuations in order to gain information about the underlying mechanism of the noise. Such studies may allow for optimizing the structures with respect to their noise level, *i.e.*, a reduction of the parameter γ*_H_*. The inset of Figure 5 shows voltage noise PSDs taken at currents *I* = 38.8 μA and *I* = 0. As required, the latter spectrum is white and represents the noise background of our experimental setup. The horizontal line roughly corresponds to the thermal (Johnson) noise *S_V_* = 4*k*_B_*TR* resulting from the sample resistance. Thick solid lines are fits to the spectra *S* ∝ 1/*f^α^* with a slope α = 1.03. Given the magnitude of the *1/f*-noise, which is determined by γ*_H_*, an extrapolation yields a crossing with the Johnson noise level at a frequency of *f_c_* ∼1 kHz. Thus, for *f* ≥ *f_c_* the ultimate sensitivity of the present sensor devices is only limited by their thermal noise level, *i.e.*, the signal-to-noise ratio may be improved by applying larger driving currents.

## 4. Conclusions

In summary, a new type of strain sensor device based on nanogranular metals has been demonstrated. The strain sensor was fabricated by FEBID of the precursor [MeCpPt(Me)_3_]. The sensitivity of the strain sensor can be tuned by changing the conductivity of the deposit leading to a maximum in the gauge factor. In contrast to the established process for the fabrication of piezoresistive sensors, that requires a complex fabrication procedures, our fabrication process is a single-step maskless procedure, making it readily usable for rapid prototyping. In addition, the high resolution of the focused electron-beam induced deposition technique allows for easy downscaling of sensor structures to below 100 nm. At the same time the sensor resistance can be adjusted to be in the several hundred Ohms to several kOhms regime at maximum sensitivity. Another advantage is the ability to monitor the process of sensor deposition *in situ*, having full control over the properties of the sensor device during the fabrication. The methodology presented here promises to be suitable for a wide area of applications, ranging from simple strain and stress measurements to advanced applications in NEMS devices and biomedical applications. Especially the immanent scalability of the FEBID process towards structures in the 10 nm range opens up a new pathway towards highly miniaturized sensor elements in conjunction with a relatively simple fabrication procedure. The process also enables the deposition of the sensor material on nearly every smooth surface in even complex geometries making real 3D applications feasible. Based on recent advances in the theory of electronic transport in nano-granular metals a systematic approach is available for further optimization of the sensitivity of strain sensor materials based on this material class. For a reduction of the grain size of the metallic nanoparticles as well as insulating matrices with small dielectric constants gauge factors larger than 50 are predicted.
